# Supplementary material for: Stochastic chain termination in bacterial pilus assembly
Source: Nat Commun. 2023 Nov 24;14:7718. doi: 10.1038/s41467-023-43449-y (PMC10673952; doi:10.1038/s41467-023-43449-y)
Supplement: Supplementary file 1 — Supplementary Information [file 41467_2023_43449_MOESM1_ESM.pdf]

Supplementary Information

Stochastic chain termination in bacterial pilus assembly

Christoph Giese,[1,*] Chasper Puorger,[1,2] Oleksandr Ignatov,[1,3] Zuzana Bečárová,[1] Marco E. Weber,[1,4] Martin A. Schärer,[1,5] Guido Capitani[5,6] and Rudi Glockshuber[1]

[1]Institute of Molecular Biology and Biophysics, Department of Biology, ETH Zurich, 8093 Zurich, Switzerland

[2]Present address: Institute for Chemistry and Bioanalytics, University of Applied Sciences and Arts Northwestern Switzerland, 4132 Muttenz, Switzerland

[3]Present address: V.I. Grishchenko Clinic of Reproductive Medicine, Blahovishchenska st.25, 61052 Kharkiv, Ukraine

[4]Present address: Laboratory of Physical Chemistry, Department of Chemistry and Applied Biosciences, ETH Zurich, 8093 Zurich, Switzerland

[5]Laboratory of Biomolecular Research, Paul Scherrer Institute, 5232 Villigen-PSI, Switzerland

[6]Deceased May 2, 2017

[*]Correspondence: giesec@mol.biol.ethz.ch

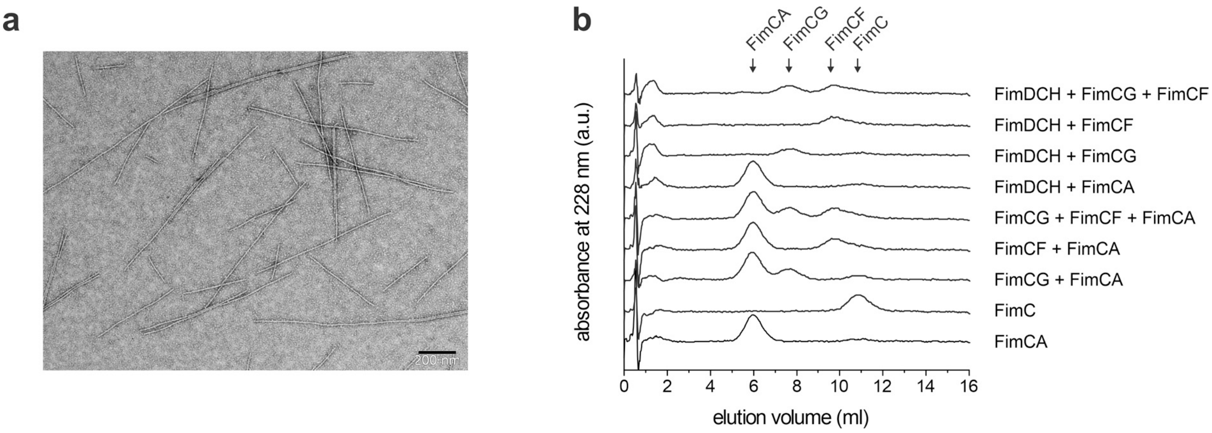

Supplementary Figure 1. Control experiments regarding the reconstitution of type 1 pilus assembly.
a) Negative-stain electron micrograph of pilus rods assembled *in vitro* by FimD. FimDCH (0.2 µM) was incubated with a 20-fold molar excess of FimCF for 5 min at 37 °C. FimCA was added to a final concentration of 10 µM resulting in a 100-fold excess over FimD, and the reaction incubated further until completion (scale bar = 200 nm).
b) Cation exchange chromatography profiles of control reactions at 37 °C proved that changes in FimCA and FimC peak intensity reflected FimD-catalyzed pilus rod assembly only and were not caused by other reactions, for instance between FimCA and FimCG/FimCF. Reactions including FimDCH and FimCG/FimCF were first preincubated for 5 min before addition of buffer and additional incubation for 10 min. All other reactions were analyzed after 10 min of incubation. Concentrations were 0.25 µM FimDCH, 2 µM FimCG and/or FimCF, 5 µM FimCA and 5 µM FimC. Source data are provided as a Source Data file.

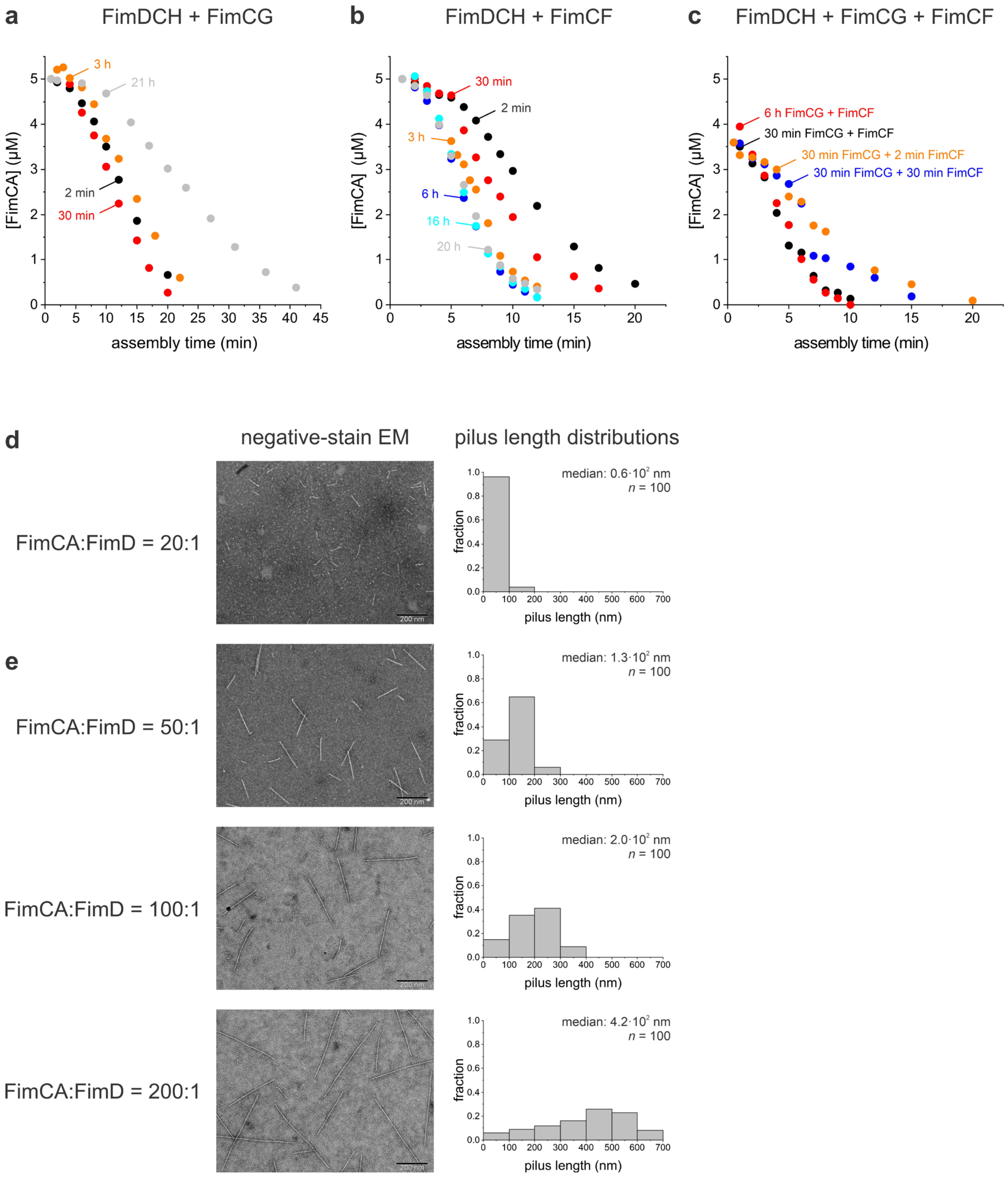

Supplementary Figure 2. Optimization of reaction conditions for type 1 pilus rod assembly *in vitro* at pH 8.0 and 23 °C.

a) Kinetics of FimD-catalyzed FimA assembly measured after activation of 0.35 µM FimDCH by 2.8 µM FimCG for the indicated periods of time. Initial concentrations of FimDCH, FimCG and FimCA in the assembly reaction were 0.25, 2 and 5 µM, respectively.

b) Kinetics of FimD-catalyzed FimA assembly measured after activation of 0.35 µM FimDCH by 2.8 µM FimCF for the indicated periods of time. Initial concentrations of FimDCH, FimCF and FimCA in the assembly reaction were 0.25, 2 and 5 µM, respectively.

c) Kinetics of FimD-catalyzed FimA assembly measured after activation of FimDCH by sequential addition of FimCG and FimCF (shown in orange and blue) or by simultaneous co-incubation of all three

complexes (shown in black and red) for the indicated periods of time. For sequential activation, FimCG and FimCF were used at 2.8 µM each, corresponding to an 8-fold excess of FimCG over FimDCH in the first, and a 14-fold excess of FimCF over FimDCH in the second step of the reaction. Initial concentrations of FimDCH, FimCG, FimCF and FimCA in the pilus rod assembly reaction were 0.18, 1.4, 2.5 and 3.6 µM, respectively. For simultaneous FimDCH activation, FimDCH, FimCG and FimCF were used at 0.35 and 2.8 µM, respectively. Initial concentrations of FimDCH, FimCG, FimCF and FimCA in the pilus rod assembly reaction were 0.18, 1.4, 1.4 and 3.6 µM, respectively.
Kinetics in (a), (b) and (c) were normalized using the known initial FimCA concentration and the peak area of the first data point.
d) and e) Negative-stain electron micrographs and length distributions of pilus rods assembled using a 20-, 50-, 100- or 200-fold excess of FimCA over FimD. FimDCH (0.35 µM) was activated by preincubation with an 8-fold excess of both FimCG and FimCF for 30 min. FimCA was added to 3.6, 9, 18 or 36 µM and samples analyzed after 20 min of assembly (scale bar = 200 nm). For pilus length distributions, the lengths of 100 pili were determined.
Source data are provided as a Source Data file.

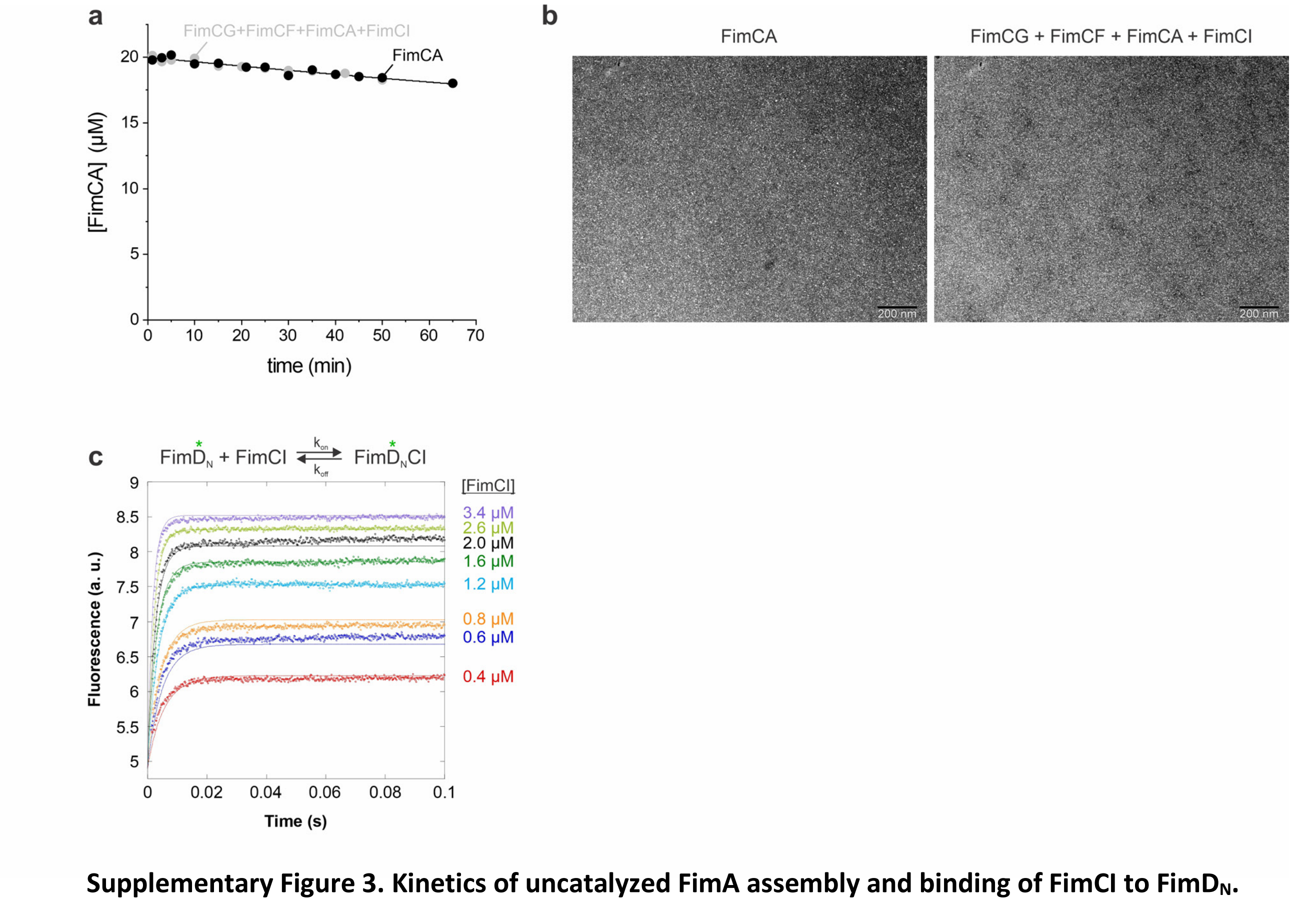

Supplementary Figure 3. Kinetics of uncatalyzed FimA assembly and binding of FimCI to FimD$_N$.

a) Kinetics of uncatalyzed FimA assembly. FimCA (20 µM) was either incubated alone or in presence of 0.8 µM FimCG, 0.8 µM FimCF and 1 µM FimCI for 65 min at 23 °C. Both data sets were globally fitted according to an irreversible second-order dimerization reaction (solid line) yielding $k_{(A\text{-}A)uncat}$ = 0.73 ± 0.02 M^{-1} s^{-1}.

b) Negative-stain electron micrographs of samples from the uncatalyzed FimA assembly reactions shown in (a), taken after 65 min of assembly (scale bar = 200 nm).

c) Binding kinetics for fluorescently labeled FimD$_N$ and FimCI measured by stopped-flow fluorescence spectroscopy at pH 8.0 and 23 °C (green asterisks indicate the fluorophore). Initial concentrations were 0.4 µM FimD$_N$ and 0.4, 0.6, 0.8, 1.2, 1.6, 2.0, 2.6 and 3.4 µM FimCI. The solid lines represent a global fit of the data according to a second-order binding and first-order dissociation reaction yielding k_{on} = 1.45 · 10^8 M^{-1} s^{-1} and k_{off} = 94 s^{-1}.

Source data are provided as a Source Data file.

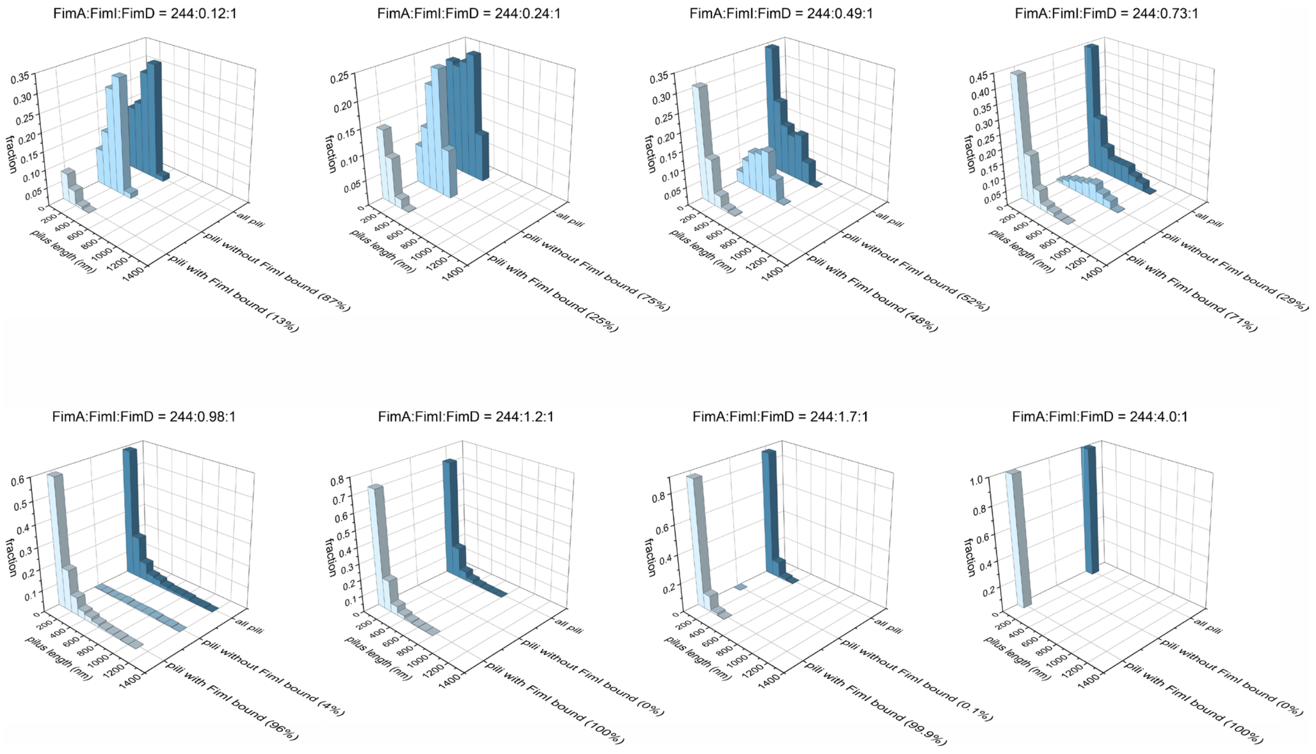

Supplementary Figure 4. FimI-terminated pili are predominantly short.
Pilus length distributions obtained after simulating type 1 pilus rod assembly in the presence of different FimCI concentrations. Each panel shows the distribution comprising all pili (in blue) together with the two underlying distributions of FimI-terminated pilus rods (in gray) and non-terminated rods (in light-blue). For each simulation, the fractions of FimI-terminated and non-terminated pilus rods are given in parentheses. Ratios between FimCA, FimCI and FimD are indicated above each panel. Source data are provided as a Source Data file.

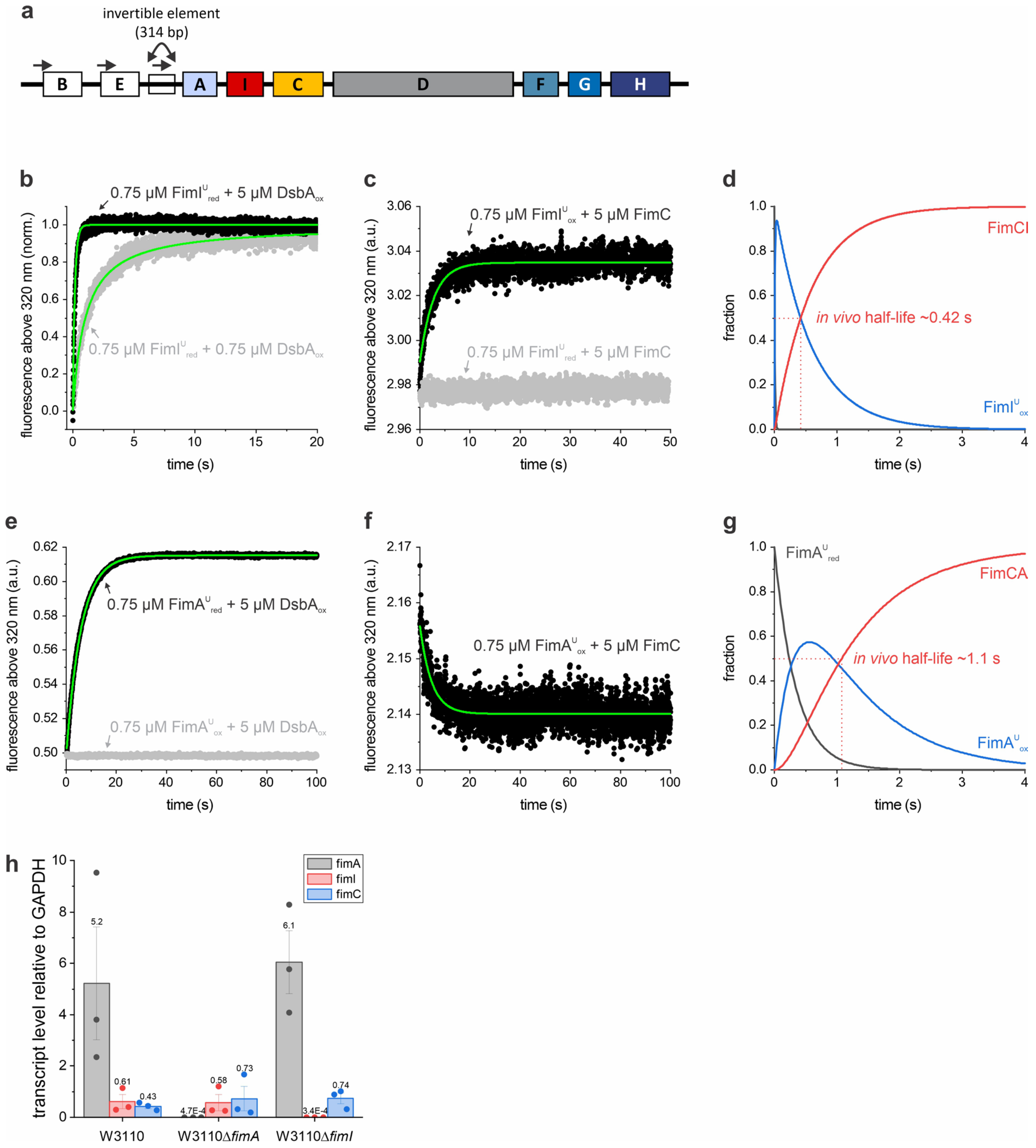

Supplementary Figure 5. Type 1 pilus gene cluster, kinetics of oxidation and folding of FimI and FimA measured by stopped-flow fluorescence spectroscopy at pH 8.0 and 25 °C and transcript levels of *fimA*, *fimI* and *fimC*.

a) Schematic representation of the type 1 pilus gene cluster[1]. Promoter regions are indicated by horizontal arrows. Expression of the *fimA*, *fimI*, *fimC*, *fimD*, *fimF*, *fimG* and *fimH* genes is controlled by a promoter located upstream of *fimA* within a 314 bp-long invertible DNA element[2,3]. The orientation of this element can be switched by two recombinases, FimB and FimE, encoded upstream of the element[4].

b) Kinetics of DsbA-catalyzed disulfide bond formation in reduced, unfolded FimI ($FimI^{U}_{red}$) recorded using identical initial concentrations of oxidized DsbA ($DsbA_{ox}$) and $FimI^{U}_{red}$ (0.75 µM each) or under pseudo-first order conditions with a 6.7-fold excess of $DsbA_{ox}$ over $FimI^{U}_{red}$. After normalization, both

datasets were globally fitted according to an irreversible, second-order reaction (green line). The rate constant for oxidation and the initial $\mathrm{FimI}^{\mathrm{U}}_{\mathrm{red}}$ concentration were shared among the datasets.

c) The kinetic of FimC-catalyzed folding of oxidized, unfolded FimI ($\mathrm{FimI}^{\mathrm{U}}_{\mathrm{ox}}$, 0.75 µM) was recorded under pseudo-first order conditions (shown in black). The solid line corresponds to a fit according to a single-exponential function. FimC specifically recognized disulfide-intact, unfolded FimI as no fluorescence change was detected when reduced, unfolded FimI was mixed with FimC (shown in gray).

d) Simulated kinetics for oxidative folding of FimI *in vivo*. The fractions of $\mathrm{FimI}^{\mathrm{U}}_{\mathrm{red}}$, $\mathrm{FimI}^{\mathrm{U}}_{\mathrm{ox}}$ and native FimCI complex were calculated using the rate constants for DsbA-catalyzed oxidation and FimC-catalyzed folding determined in (b) and (c), the experimentally determined periplasmic concentrations of DsbA (86 µM) and FimC (23 µM) and assuming pseudo-first order conditions for FimI, i.e. that DsbA and FimC are in excess over $\mathrm{FimI}^{\mathrm{U}}_{\mathrm{red}}$ and $\mathrm{FimI}^{\mathrm{U}}_{\mathrm{ox}}$ in the periplasm, respectively. The resulting, theoretical *in vivo* half-life for formation of the native FimCI complex is indicated.

e) Kinetic of DsbA-catalyzed disulfide bond formation in reduced, unfolded FimA ($\mathrm{FimA}^{\mathrm{U}}_{\mathrm{red}}$, 0.75 µM) recorded under pseudo-first order conditions (shown in black). The solid line corresponds to a fit according to a single-exponential function. No reaction occurred when oxidized, unfolded FimA ($\mathrm{FimA}^{\mathrm{U}}_{\mathrm{ox}}$) was mixed with oxidized DsbA (shown in gray).

f) Kinetic of FimC-catalyzed folding of $\mathrm{FimA}^{\mathrm{U}}_{\mathrm{ox}}$ (0.75 µM) recorded under pseudo-first order conditions and fitted according to a single-exponential function (solid line).

g) Simulated kinetics for oxidative folding of FimA *in vivo*. The fractions of $\mathrm{FimA}^{\mathrm{U}}_{\mathrm{red}}$, $\mathrm{FimA}^{\mathrm{U}}_{\mathrm{ox}}$ and native FimCA complex were calculated using the rate constants for DsbA-catalyzed oxidation and FimC-catalyzed folding determined in (e) and (f), the experimentally determined periplasmic concentrations of DsbA (86 µM) and FimC (23 µM) and assuming pseudo-first order conditions for FimA. The resulting, theoretical *in vivo* half-life for formation of the native FimCA complex is indicated.

h) Transcript levels of *fimA*, *fimI* and *fimC* in *E. coli* W3110, W3110Δ*fimA* and W3110Δ*fimI* cells determined by real-time PCR relative to the transcript level of GAPDH. Data are means ± s.e. of three biological replicates (shown as individual data points) with three technical replicates each.
Source data are provided as a Source Data file.

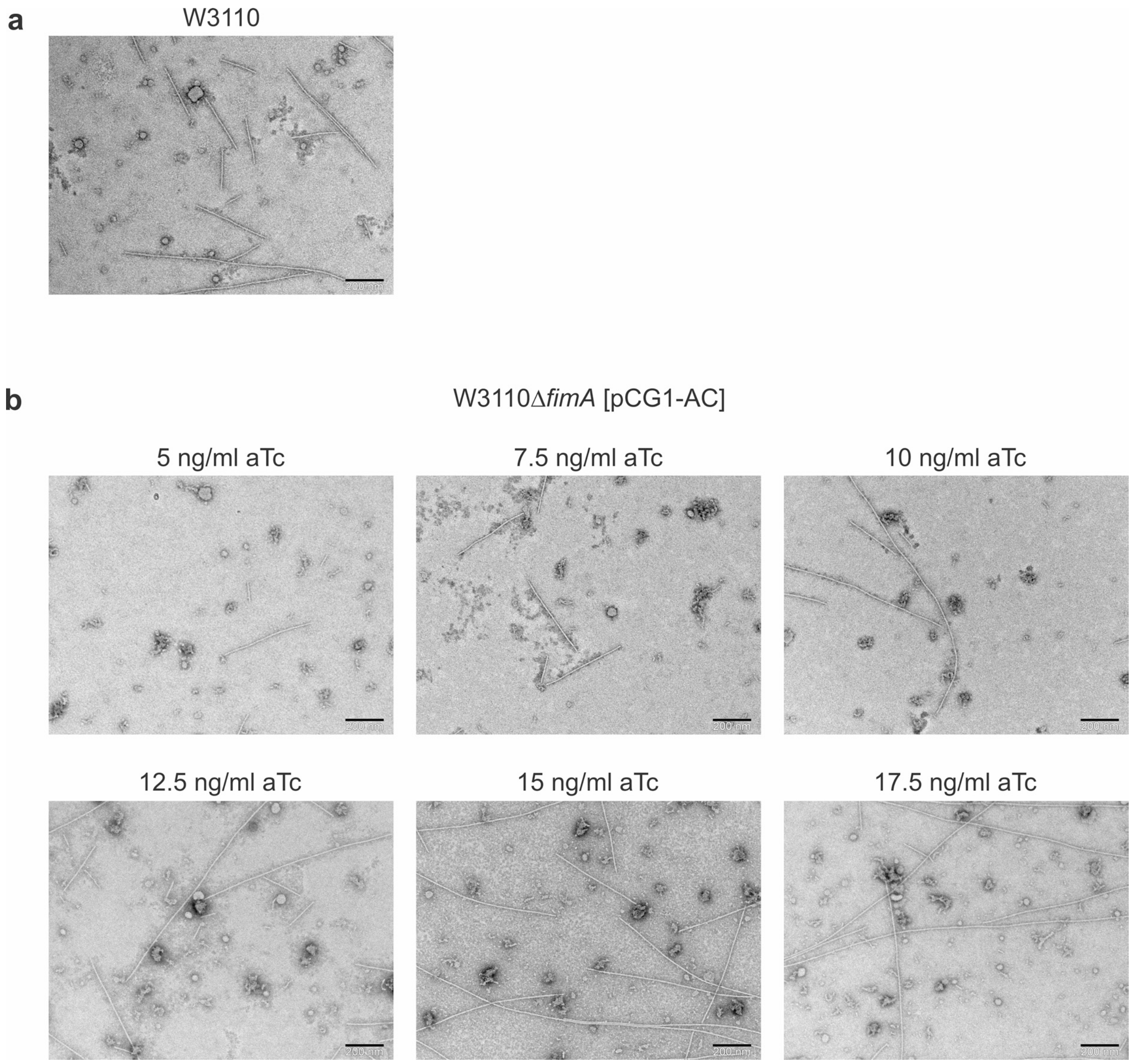

Supplementary Figure 6. Negative-stain electron micrographs of pili released from cells.
Pili released from a) *E. coli* W3110 cells or b) W3110Δ*fimA* cells harboring pCG1-AC and grown in presence of the indicated anhydrotetracycline (aTc) concentrations. Scale bar = 200 nm.

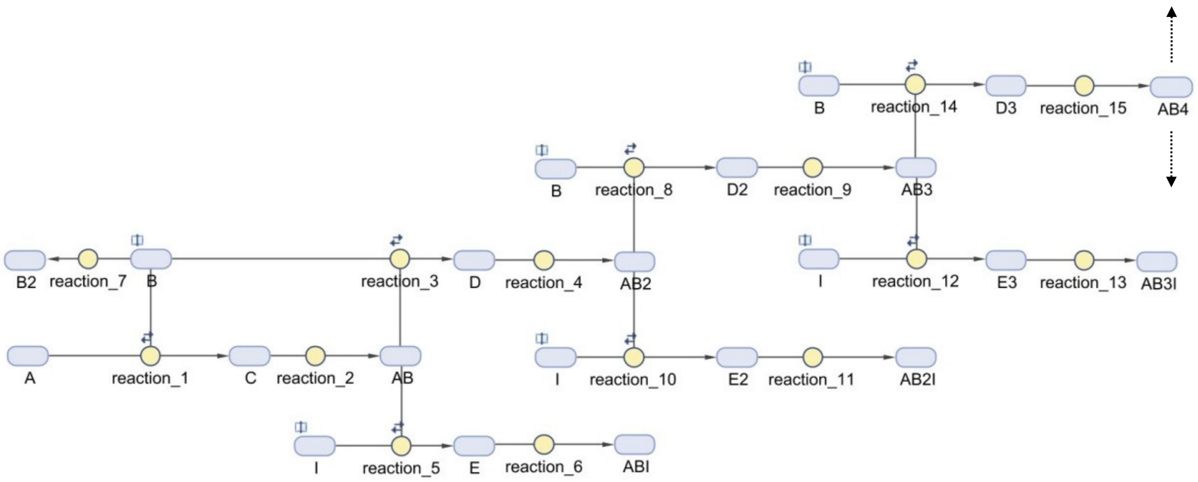

Supplementary Figure 7. Stochastic simulation of pilus rod assembly and assembly termination. Schematic of part of the pilus rod assembly mechanism used for Monte Carlo simulations in MATLAB and SimBiology. Each species occurring during the assembly reaction was represented by a dedicated pool. For example, species A, B and I denote activated FimDCH, FimCA and FimCI, respectively; species AB, AB2, AB3 and AB4 denote pili with 1, 2, 3 or 4 FimA monomers incorporated and ABI, AB2I and AB3I represent pili with 1, 2 and 3 FimA monomers that were terminated by incorporation of FimI. Dotted arrows indicate the direction of the continuing reaction.

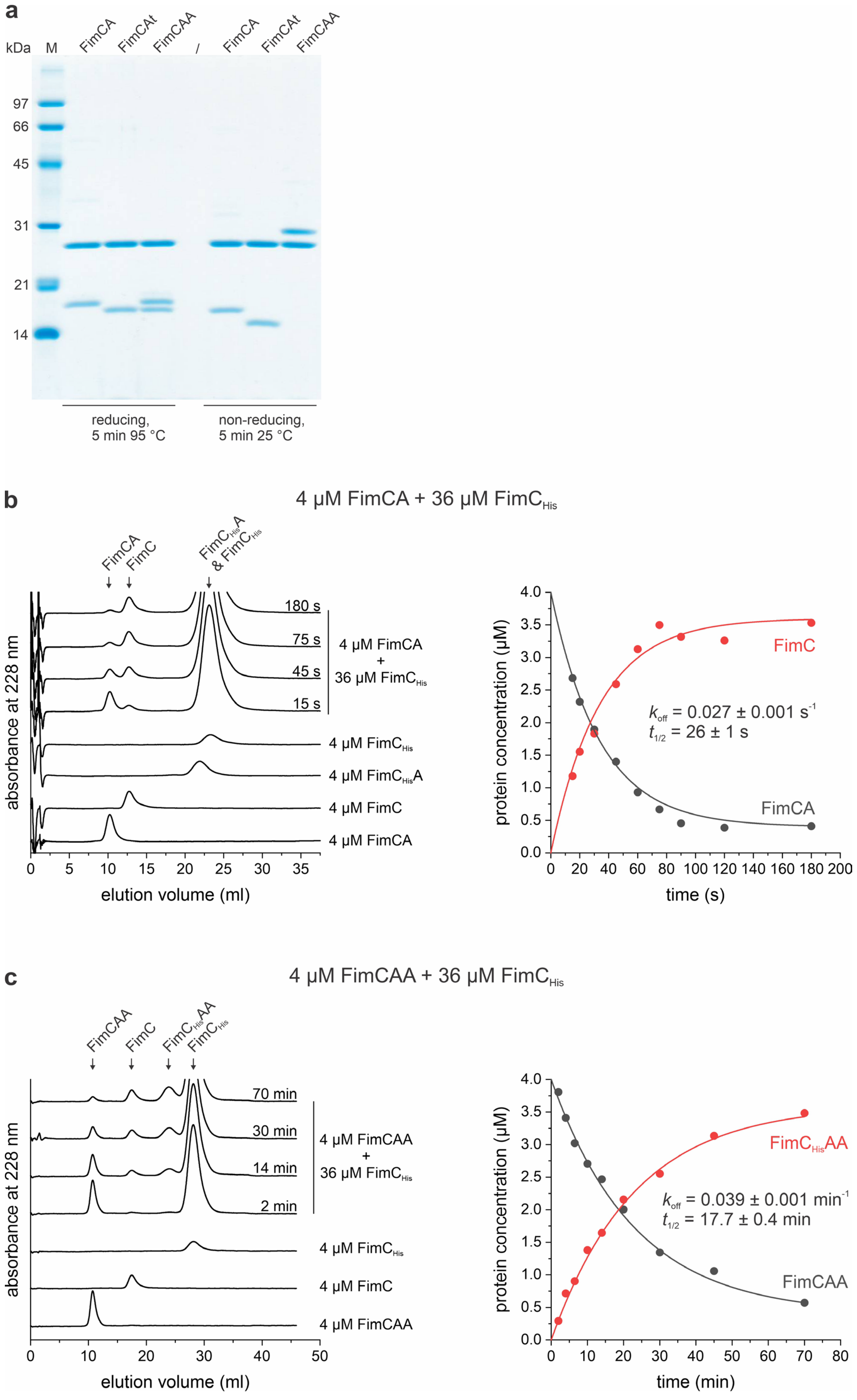
a
kDa M
FimCA
FimCAt
FimCAA
/
FimCA
FimCAt
FimCAA
97
66
45
31
21
14
reducing, 5 min 95 °C
non-reducing, 5 min 25 °C
b
4 µM FimCA + 36 µM FimC$_{His}$
FimCA
FimC
FimC$_{His}$A & FimC$_{His}$
180 s
75 s
45 s
15 s
4 µM FimCA + 36 µM FimC$_{His}$
4 µM FimC$_{His}$
4 µM FimC$_{His}$A
4 µM FimC
4 µM FimCA
absorbance at 228 nm
elution volume (ml)
protein concentration (µM)
time (s)
FimC
FimCA
k_{off} = 0.027 ± 0.001 s^{-1}
$t_{1/2}$ = 26 ± 1 s
c
4 µM FimCAA + 36 µM FimC$_{His}$
FimCAA
FimC
FimC$_{His}$AA
FimC$_{His}$
70 min
30 min
14 min
2 min
4 µM FimCAA + 36 µM FimC$_{His}$
4 µM FimC$_{His}$
4 µM FimC
4 µM FimCAA
absorbance at 228 nm
elution volume (ml)
protein concentration (µM)
time (min)
FimC$_{His}$AA
FimCAA
k_{off} = 0.039 ± 0.001 min^{-1}
$t_{1/2}$ = 17.7 ± 0.4 min

Supplementary Figure 8. Kinetics of FimC dissociation from FimCA and FimCAA at 37 °C.

a) Coomassie-stained polyacrylamide-SDS gel of purified FimC-FimA (FimCA), FimC-FimAt (FimCAt) and the ternary FimC-FimA-FimAt (FimCAA) complex (25 pmol each). Samples containing SDS were incubated for five minutes at 95 °C in presence of DTT (reducing condition) or at 25 °C in absence of DTT (non-reducing condition). Under the former conditions, the FimA-FimAt dimer in FimCAA is fully denatured and dissociated, giving rise to two discrete bands corresponding to FimA and FimAt. Omitting the heating step and DTT leaves the FimA-FimAt complex intact and results in a single band for FimA-FimAt with an apparent molecular mass of ~30 kDa.

b), c) Kinetics of FimC dissociation from FimCA (b) and FimCAA (c) at 37 °C. After mixing 4 µM FimCA or FimCAA with 36 µM $FimC_{His}$, samples taken after different reaction times were analyzed by analytical cation exchange chromatography. Representative elution profiles together with controls are shown in the left panels. The kinetics for the relaxation to the new equilibrium are shown in the right panels, solid lines are fits of the data to a single-exponential function. As it was not possible to resolve $FimC_{His}$ and $FimC_{His}A$ for the reaction including FimCA, we here quantified formation of free FimC instead of $FimC_{His}A$.

Source data are provided as a Source Data file.

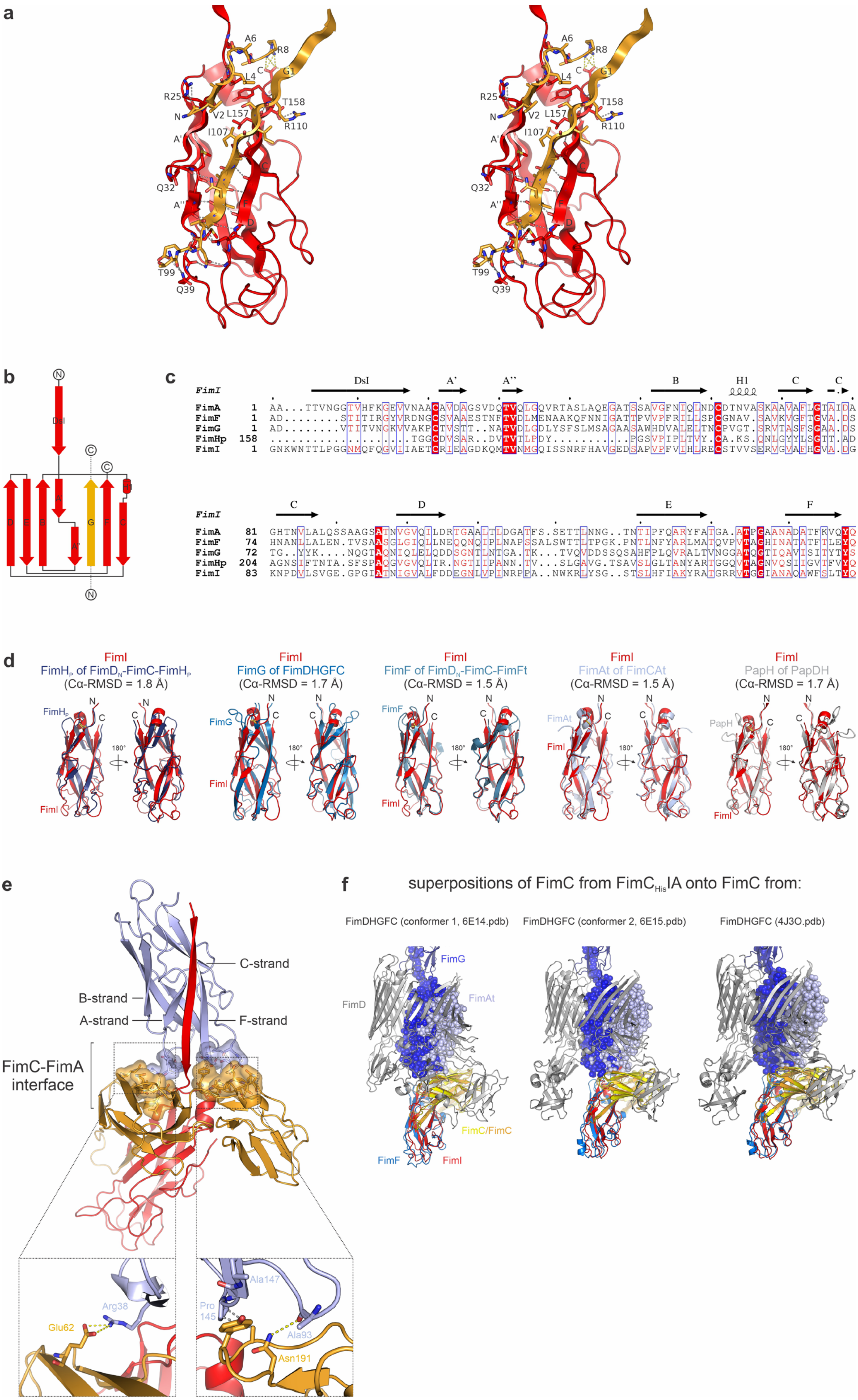
a
A6
R8
C
G1
L4
R25
N
V2
L157
T158
R110
I107
A'
Q32
A''
T99
Q39
b
DsI
c
FimI
DsI
A'
A''
B
H1
C
FimA 1 AA...TTVNGGTVHFKGEVVNAACAVDAGSVDQTVQLGQVRTASLAQEGATSSAVGFNIQLNDCDTNVASKAAVAFLGTAIDA
FimF 1 AD........STITIRGYVRDNGCSVAAESTNFTVDLMENAAKQFNNIGATTPVVPFRILLSPCGNAV.SAVKVGFTGVA.DS
FimG 1 AD........VTITVNGKVVAKPCTVSTT..NATVDLGDLYSFSLMSAGAASAWHDVALELTNCPVGT.SRVTASFSGAA.DS
FimHp 158 ...................TGGCDVSAR..DVTVTLPDY...........PGSVPIPLTVY.CA.KS.QNLGYYLSGTT.AD
FimI 1 GNKWNTTLPGGNMQFQGVIIAETCRIEAGDKQMTVNMGQISSNRFHAVGEDSAPVPFVIHLRECSTVVSERVGVAFHGVA.DG
FimI
C
D
E
F
FimA 81 GHTNVLALQSSAAGSATNVGVQILDRTGAALTLDGATFS.SETTLNNG..TNTIPFQARYFATGA.ATPGAANADATFKVQYQ
FimF 74 HNANLLALEN.TVSAASGLGIQLLNEQQNQIPLNAPSSALSWTTLTPGK.PNTLNFYARLMATQVPVTAGHINATATFTLEYQ
FimG 72 TG..YYK....NQGTAQNIQLELQDDSGNTLNTGA.TK...TVQVDDSSQSAHFPLQVRALTVNGGATQGTIQAVISITYTYS
FimHp 204 AGNSIFTNTA.SFSPAQGVGVQLTR.NGTIIPANN.TV...SLGAVG.TSAVSLGLTANYARTGGQVTAGNVQSIIGVTFVYQ
FimI 83 KNPDVLSVGE.GPGIATNIGVALFDDEGNLVPINRPPA..NWKRLYSG..STSLHFIAKYRATGRRVTGGIANAQAWFSLTYQ
d
FimI
FimHP of FimDN-FimC-FimHP
(Cα-RMSD = 1.8 Å)
FimI
FimG of FimDHGFC
(Cα-RMSD = 1.7 Å)
FimI
FimF of FimDN-FimC-FimFt
(Cα-RMSD = 1.5 Å)
FimI
FimAt of FimCAt
(Cα-RMSD = 1.5 Å)
FimI
PapH of PapDH
(Cα-RMSD = 1.7 Å)
N
C
180°
FimHP
FimG
FimF
FimAt
PapH
FimI
e
C-strand
B-strand
A-strand
F-strand
FimC-FimA interface
Arg38
Glu62
Ala147
Pro 145
Ala93
Asn191
f
superpositions of FimC from FimCHisIA onto FimC from:
FimDHGFC (conformer 1, 6E14.pdb)
FimDHGFC (conformer 2, 6E15.pdb)
FimDHGFC (4J3O.pdb)
FimG
FimAt
FimD
FimC/FimC
FimF
FimI

Supplementary Figure 9. Structural analysis of the $FimC_{His}IA$ complex.

a) Stereo view of how FimC and FimI interact in the $FimC_{His}IA$ complex. FimC is in gold, FimI in red. The N-terminus of FimC, the C-terminus of FimI, and selected residues and β-strands are labelled. For clarity, only FimC residues 1-8 and 99-116 are shown, FimAt is omitted. Hydrogen bonds and salt bridge are indicated by dashed gray and yellow lines.

b) Topology diagram of the FimI fold as found in the structure of the $FimC_{His}IA$ complex. β-strands are depicted as arrows, α-helices as cylinders. The FimC donor strand is shown in gold and runs parallel to the C-terminal F-strand of FimI.

c) Structure-based sequence alignment of all type 1 pilus subunits. Similar residues are in blue boxes and highlighted in red, identical residues are shown in white on red background. The secondary structure elements of FimI are displayed on top of the alignment.

d) Structural superposition of FimI of the $FimC_{His}IA$ complex onto $FimH_P$, FimG, FimF, FimAt and PapH of the $FimD_N$-FimC-$FimH_P$ (1ZE3.pdb), FimDHGFC (4J3O.pdb), $FimD_N$-FimC-$FimF_t$ (3BWU.pdb), FimCAt (4DWH.pdb) and PapDH complex (2J2Z.pdb), respectively.

e) Crystal structure of the $FimC_{His}IA$ complex shown in cartoon representation with the 351 $Å^2$ interface formed between FimC and FimAt shown in surface representation. FimC is in gold, FimI in red and FimAt in light blue. Details of the FimC-FimAt interaction are shown in boxes at the bottom of the panel. The salt bridge, hydrogen bond and hydrophobic interactions are indicated as dashed yellow and gray lines.

f) Structural superposition of FimC from the $FimC_{His}IA$ complex onto FimC from the FimDHGFC complex. All proteins are shown in cartoon representation except for FimG and FimAt, which are shown as spheres. FimD is in gray, FimG in blue, FimF in marine, FimC in yellow/gold, FimI in red and FimAt in light blue. Note that while FimF and FimI superimpose well, FimAt clashes with FimD and does not align with FimG inside the pore of FimD.

Supplementary Table 1. Parameters of pilus length distributions obtained after *in vivo* pilus assembly.

E. coli strain		mean ± SD (nm)	median (nm)	mean ± SD of 5% longest pili (µm)
W3110		$(3.0 \pm 2.3)\cdot10^2$	$2.4\cdot10^2$	0.95 ± 0.21
W3110Δ*fimA* [pCG1-AC]	+ 5 ng/ml aTc	$(1.0 \pm 1.2)\cdot10^2$	$0.7\cdot10^2$	0.50 ± 0.32
	+ 7.5 ng/ml aTc	$(2.1 \pm 2.2)\cdot10^2$	$1.4\cdot10^2$	0.95 ± 0.26
	+ 10 ng/ml aTc	$(2.6 \pm 3.8)\cdot10^2$	$1.4\cdot10^2$	1.62 ± 0.53
	+ 12.5 ng/ml aTc	$(3.3 \pm 3.5)\cdot10^2$	$2.1\cdot10^2$	1.35 ± 0.36
	+ 15 ng/ml aTc	$(4.8 \pm 5.8)\cdot10^2$	$2.6\cdot10^2$	2.36 ± 0.61
	+ 17.5 ng/ml aTc	$(8.1 \pm 10.5)\cdot10^2$	$4.0\cdot10^2$	3.96 ± 0.54

Pili were released from *E. coli* W3110 or W3110Δ*fimA* [pCG1-AC] grown in presence of the indicated anhydrotetracycline (aTc) concentrations and parameters obtained from length measurements of 200 pili each. SD denotes the standard deviation of the mean.

Supplementary Table 2. X-ray data collection and refinement statistics for structure determination of the $FimC_{His}It$, $FimD_NCI$ and $FimC_{His}IA$ complexes.

	**$FimC_{His}It$**	**$FimD_NCI$**	**$FimC_{His}IA$**
pdb entry	7B0W	7B0X	6SWH
**Data collection**			
Space group	$P3_121$	$P2_12_12_1$	$P6_1$
Cell dimensions			
a, *b*, *c* (Å)	166.22, 166.22, 51.44	35.24, 104.16, 132.16	198.35, 198.35, 87.56
α, β, γ (°)	90.0, 90.0, 120.0	90.0, 90.0, 90.0	90.0, 90.0, 120.0
Resolution (Å)	50 – 1.75 (1.8 – 1.75)*	50 – 1.7 (1.7 – 1.74)*	22 – 2.8 (3.0 – 2.8)*
No. reflections	279213 (21175)*	226688 (16718)*	282336 / (45948)*
Redundancy	3.4 (3.5)*	4.2 (4.2)*	5.9 (5.1)*
Completeness (%)	99.1 (99.5)*	99.1 (99.6)*	99.3 (99.2)*
$I/\sigma I$	11.6 (1.26)*	11.9 (1.1)*	21.27 (3.07)*
$CC_{1/2}$ (%)	99.8 (50.3)*	99.9 (44.9)*	99.9 (80.0)*
R_{meas} (%)	8.6 (127.8)*	9.3 (144.1)*	7.2 (64.3)*
**Refinement**			
Resolution (Å)	48.4 – 1.75	48.5 – 1.7	19.89 – 2.80
No. reflections (test set)	81369 (977)	54117 (1083)	48217 (1010)
R_{work} / R_{free} (%)	17.1 / 18.7	20.4 / 25.2	17.05 / 20.70
Ramachandran plot analysis			
Favored region (%)	98.5	98.7	96.39
Outliers (%)	0.00	0.00	0.00
No. of atoms			
Protein	2652	3663	7206
Ligand/ion	38	4	11
Water	441	418	163
B-factors ($Å^2$)			
Protein	33.2	34.7	81.0
Ligand/ion	43.2	37.6	83.6
Water	40.8	51.8	62.8
R.m.s. deviations			
Bond lengths (Å)	0.006	0.007	0.0046
Bond angles (°)	0.792	1.032	0.670

Data was collected from a single crystal.
* Values in parentheses are for the highest-resolution shell.

Supplementary References

1. Schwan, W. R. Regulation of fim genes in uropathogenic Escherichia coli. *World J Clin Infect Dis* **1**, 17-25 (2011).
2. Abraham, J. M., Freitag, C. S., Clements, J. R. & Eisenstein, B. I. An invertible element of DNA controls phase variation of type 1 fimbriae of Escherichia coli. *Proc Natl Acad Sci U S A* **82**, 5724-5727 (1985).
3. Olsen, P. B. & Klemm, P. Localization of promoters in the fim gene cluster and the effect of H-NS on the transcription of fimB and fimE. *FEMS Microbiol Lett* **116**, 95-100 (1994).
4. Klemm, P. Two regulatory fim genes, fimB and fimE, control the phase variation of type 1 fimbriae in Escherichia coli. *EMBO J* **5**, 1389-1393 (1986).
