# Supplementary material for: Stochastic chain termination in bacterial pilus assembly
Source: Nat Commun. 2023 Nov 24;14:7718. doi: 10.1038/s41467-023-43449-y (PMC10673952; doi:10.1038/s41467-023-43449-y)
Supplement: Supplementary file 4 — Reporting Summary [file 41467_2023_43449_MOESM4_ESM.pdf]

nature portfolio

Corresponding author(s): Christoph Giese

Last updated by author(s): Oct 27, 2023

Reporting Summary

Nature Portfolio wishes to improve the reproducibility of the work that we publish. This form provides structure for consistency and transparency in reporting. For further information on Nature Portfolio policies, see our Editorial Policies and the Editorial Policy Checklist.

Statistics

For all statistical analyses, confirm that the following items are present in the figure legend, table legend, main text, or Methods section.

n/a	Confirmed	
☐	☒	The exact sample size (n) for each experimental group/condition, given as a discrete number and unit of measurement
☐	☒	A statement on whether measurements were taken from distinct samples or whether the same sample was measured repeatedly
☒	☐	The statistical test(s) used AND whether they are one- or two-sided<br>*Only common tests should be described solely by name; describe more complex techniques in the Methods section.*
☒	☐	A description of all covariates tested
☒	☐	A description of any assumptions or corrections, such as tests of normality and adjustment for multiple comparisons
☐	☒	A full description of the statistical parameters including central tendency (e.g. means) or other basic estimates (e.g. regression coefficient) AND variation (e.g. standard deviation) or associated estimates of uncertainty (e.g. confidence intervals)
☒	☐	For null hypothesis testing, the test statistic (e.g. F, t, r) with confidence intervals, effect sizes, degrees of freedom and P value noted<br>*Give P values as exact values whenever suitable.*
☒	☐	For Bayesian analysis, information on the choice of priors and Markov chain Monte Carlo settings
☒	☐	For hierarchical and complex designs, identification of the appropriate level for tests and full reporting of outcomes
☒	☐	Estimates of effect sizes (e.g. Cohen's d, Pearson's r), indicating how they were calculated

Our web collection on statistics for biologists contains articles on many of the points above.

Software and code

Policy information about availability of computer code

Data collection	Cary WinUV 3.00, Unicorn 5.11, Pro-Data SX 2.4.0, Morgagni 268 3.0, iTEM 5.2
Data analysis	PeakFit 4.12, Origin 9.1, DynaFit 4.07.135, Fiji 1.53c, TrakEM2 1.0a, CorelDRAW 22.0.0.412, SDS 2.3, XDS, Phenix 1.18.2, CCP4 7.0, WinCoot 0.8.6.1, PyMOL 2.5.3, Castp webserver, PISA webserver, MATLAB R2019b, SimBiology 5.9

For manuscripts utilizing custom algorithms or software that are central to the research but not yet described in published literature, software must be made available to editors and reviewers. We strongly encourage code deposition in a community repository (e.g. GitHub). See the Nature Portfolio guidelines for submitting code & software for further information.

Data

Policy information about availability of data

All manuscripts must include a data availability statement. This statement should provide the following information, where applicable:

- Accession codes, unique identifiers, or web links for publicly available datasets
- A description of any restrictions on data availability
- For clinical datasets or third party data, please ensure that the statement adheres to our policy

Atomic coordinates were deposited in the Protein Data Bank (PDB) with accession codes 6SWH (FimCHisIA), 7B0W (FimCHisIt) and 7B0X (FimDNCI). Previously solved structures used in this study have PDB accession codes 4DWH, 1ZE3 and 3SQB. Source data are provided with this paper.

Research involving human participants, their data, or biological material

Policy information about studies with human participants or human data. See also policy information about sex, gender (identity/presentation), and sexual orientation and race, ethnicity and racism.

Reporting on sex and gender	N/A
Reporting on race, ethnicity, or other socially relevant groupings	N/A
Population characteristics	N/A
Recruitment	N/A
Ethics oversight	N/A

Note that full information on the approval of the study protocol must also be provided in the manuscript.

Field-specific reporting

Please select the one below that is the best fit for your research. If you are not sure, read the appropriate sections before making your selection.

[x] Life sciences [ ] Behavioural & social sciences [ ] Ecological, evolutionary & environmental sciences

For a reference copy of the document with all sections, see nature.com/documents/nr-reporting-summary-flat.pdf

Life sciences study design

All studies must disclose on these points even when the disclosure is negative.

Sample size	For analysis of experimental pilus length distributions, generally, the length of 200 pili was measured (Figures 2B, 3B, 4C). This sample size was chosen because it was sufficiently large to accurately describe the populations. For example, using a larger sample size of n=300 for pili of E. coli W3110 cells in Figure 4C resulted in identical median values compared to n=200 (given that median values could be reliably determined with a precision of 10 nm). In one case (Figure S2), smaller sample sizes (n=100) were sufficient to confirm expected trends in the distributions; obtaining highly accurate median values was not the objective here. For qPCR experiments, samples from three biological replicates, with three technical replicates each, were analyzed.
Data exclusions	Monte Carlo simulations of pilus rod assembly reactions also resulted in very short pili with lengths < 15 nm. For comparing the simulated to the experimental pilus length distributions (Figures 3B, 3C), pili shorter than 15 nm were excluded from the simulated data sets as it was not possible to distinguish such short pili (if present) from background in the EM micrographs of the experimental samples.
Replication	All biochemical and biophysical experiments were performed independently at least two times and could successfully be replicated. Representative results are shown.
Randomization	Randomization of samples was not relevant to the experiments of this study, which were performed under controlled conditions in vitro with samples of known and defined composition.
Blinding	Blinding of investigators was not relevant to the experiments of this study, which were performed under controlled conditions in vitro with samples of known and defined composition.

Reporting for specific materials, systems and methods

We require information from authors about some types of materials, experimental systems and methods used in many studies. Here, indicate whether each material, system or method listed is relevant to your study. If you are not sure if a list item applies to your research, read the appropriate section before selecting a response.

Materials & experimental systems

n/a	Involved in the study	
[x]	[ ]	Antibodies
[x]	[ ]	Eukaryotic cell lines
[x]	[ ]	Palaeontology and archaeology
[x]	[ ]	Animals and other organisms
[x]	[ ]	Clinical data
[x]	[ ]	Dual use research of concern
[x]	[ ]	Plants

Methods

n/a	Involved in the study	
[x]	[ ]	ChIP-seq
[x]	[ ]	Flow cytometry
[x]	[ ]	MRI-based neuroimaging
